# Dietary pattern, dietary total antioxidant capacity, and dyslipidemia in Korean adults

**DOI:** 10.1186/s12937-019-0459-x

**Published:** 2019-07-13

**Authors:** Seong-Ah Kim, Hyojee Joung, Sangah Shin

**Affiliations:** 1 Department of Food and Nutrition, Chung-Ang University, Gyeonggi-do, 17546 Korea; 2 Institute of Health and Environment, Seoul National University, Seoul, 08826 Korea; 3 Graduate School of Public Health, Seoul National University, Seoul, 08826 Korea

**Keywords:** Dyslipidemia, Hypercholesterolemia, Hypertriglyceridemia, hypoHDL-cholesterolemia, Dietary pattern, Dietary total antioxidant capacity, KNHANES

## Abstract

**Background:**

Abnormal diet is considered to be an important risk factor for dyslipidemia. However, so far, most studies have focused on the association between single factors only, such as specific nutrients, foods, or dietary patterns, and dyslipidemia risk. This study aimed to examine the association of the joint interaction between dietary pattern and dietary total antioxidant capacity (TAC) with dyslipidemia.

**Methods:**

We performed a dietary pattern analysis and calculated the dietary TAC based on 24-h dietary recall (DR) data from Korea National Health and Nutrition Examination Survey (KNHANES) 2007–2012, which is representative population-based cross-sectional survey in Korea. A total of 29,624 participants aged over 19 years were included for the analysis. The number of people with hypercholesterolemia, hypertriglyceridemia, and hypoHDL-cholesterolemia was 3703, 3513, and 9802, respectively. We examined the association between the joint classifications of dietary pattern score tertiles and dietary TAC level tertiles and dyslipidemia.

**Results:**

Our results demonstrated that the “Rice & Kimchi” pattern was associated with low prevalence of hypercholesterolemia, and high prevalence of hypertriglyceridemia and hypoHDL-cholesterolemia; whereas the pattern of both “Oil, sweets, fish & other vegetables” and “Grain, bean, nuts, vegetables & fruits” were associated with low prevalence of hypertriglyceridemia. Also we demonstrated that for all dietary patterns except for the “Grain, bean, nuts, vegetables & fruits”, dietary TAC was inversely associated with hypertriglyceridemia.

**Conclusion:**

This study provides basic data for the lipid-lowering effect of dietary TAC and its interaction with dietary patterns. Further study will be needed to investigate the association between dietary TAC and dietary patterns with other diseases like metabolic syndrome, cardiovascular disease, or cancer.

**Electronic supplementary material:**

The online version of this article (10.1186/s12937-019-0459-x) contains supplementary material, which is available to authorized users.

## Background

Numerous epidemiological studies have demonstrated that dyslipidemia is a major cause of cardiovascular disease [1, 2]. Dyslipidemia is a condition characterized by abnormal lipid status (such as triglycerides [TG], cholesterol and/or phospholipids) in the blood. It comprises of elevated blood concentrations of the triad; low density lipoprotein cholesterol (LDL-C), TG, and decreased high-density lipoprotein cholesterol (HDL-C) [3]. Among these three components of dyslipidemia, elevated TG and decreased HDL-C are considered as prominent problems in Korea. The prevalence of elevated TG among Koreans rapidly increased from 10.2% in 1998 to 16.8% in 2012 [4], while that of decreased HDL-C was 26.2% in 2010 [5]. These two risk factors, elevated TG and decreased HDL-C, are commonly observed in Asian population. Recent pooled study of dyslipidemia showed that the prevalence of decreased HDL-C was 33.1% in Asian versus 27.0% in non-Asian population [6]. Various studies pointed to the fact that diet may be a possible cause of such clear difference in dyslipidemia prevalence among populations [2, 7].

Among the known risk factors of dyslipidemia, including genetic factors, hormonal abnormalities, and lifestyle factors [8], diet is considered to be one of the most important risk factors, which plays a key role in the development of dyslipidemia [9]. There are numerous evidences demonstrating the association between diet and blood lipid levels. Several studies indicated that dietary fat and cholesterol intake is associated with blood lipid levels [10, 11], while vegetables and fruits consumption is inversely associated with blood lipid levels [12, 13]. However, few studies have researched the association between overall dietary pattern and the risk of dyslipidemia.

Recently, a growing body of research has addressed the importance of phytochemicals in decreasing the risk of chronic disease. This is because phytochemicals in plants, such as flavonoids and other phenolic compounds, have antioxidant capacity which may be able to protect cells from oxidative damage caused by free radicals [14]. Oxidative stress is known to cause oxidative damage to proteins and DNA, resulting in an increased risk of cancer and cardiovascular disease [15]. Among many kinds of phytochemicals, flavonoids have been suggested to reduce the risk of several age-related chronic diseases, by protecting LDL and very low-density lipoproteins (VLDL) from oxidation [16, 17]. Antioxidant vitamins including vitamin A, C, E, and carotenoids also have beneficial health effects, by protecting LDL against oxidation and enhancing tumor surveillance through the immune system [18, 19]. However, many antioxidant compounds generally coexist in foods, therefore, it may not be appropriate to examine the effect of any single antioxidant on human health. The concept of dietary total antioxidant capacity (TAC) was then introduced, considering the synergistic interaction between antioxidants and their differential ability against oxidation [20]. TAC is the cumulative ability of all antioxidants in food and characterizes the sum of all antioxidant properties of nutrients or compounds [21]. This concept can account for the additive and synergistic interaction of dietary antioxidants rather than individual effect of single antioxidants [21]. Dietary TAC has shown beneficial effects on degenerative diseases such as cancer, cardiovascular disease, and type 2 diabetes [22–24]. However, there is insufficient evidence about the association between dietary TAC and risk of dyslipidemia.

Furthermore, among various diet-related factors influencing dyslipidemia risk, most studies have focused on the association between single factors only, including specific nutrients, foods, or dietary patterns, and dyslipidemia risk. However, since an intricate interaction exists between each diet-related factors, the study examining the synergistic effects of those factors, needed to be conducted.

In recent nutritional epidemiological studies, the dietary pattern approach has emerged and widely used to examine complicated interactional and synergistic effects of nutrients and foods, on disease [25, 26]. Dietary patterns conceptually indicate a broader picture of the overall diet; therefore, examining the dietary patterns may be more predictive of disease risk than individual nutrients or foods [25]. In particular, because antioxidants are found in a wide variety of foods, the application of the dietary pattern method is more integrated than the analysis of single food or nutrient.

Therefore, in the present study, we performed the dietary pattern analysis to identify major dietary patterns in Korean population, to compare TAC levels according to dietary patterns; and finally, to examine the association of the joint interaction between dietary pattern and TAC with dyslipidemia.

## Methods

### Study population

Study population was participants in the fourth and fifth Korea National Health and Nutrition Examination Survey (KNHANES IV and KNHANES V) (2007–2012). KNHANES is a cross-sectional, nationally representative survey in South Korea carried out by the Korea Centers for Disease Control and Prevention every three years. KNHANES consists of a health examination survey, health behavioral survey, and nutritional survey. Among 33,843 adults aged over 19 years who had participated in all three surveys, we excluded people with implausible energy intake (< 500 kcal/day or ≥ 5000 kcal/day) and insufficient information on blood samples, especially those who had missing data for total cholesterol, HDL-C, and TG. A total of 29,624 participants were finally selected for the analysis. The present study was conducted according to the guidelines laid down in the Declaration of Helsinki. All procedures for the Korea National Health and Nutrition Examination Survey were approved by the Korea Centers for Disease Control and Prevention Institutional Review Board (IRB No: 2007-02CON-04-P, 2008-04EXP-01-C, 2009-01CON-03-2C, 2010-02CON-21-C, 2011-02CON-06-C, and 2012-01EXP-01-2C), and all participants provided written informed consent.

### Anthropometric measurement and blood test

Participants’ height and body weight, measured during the health examination survey, were used to calculate the body mass index (BMI, kg/m^2^) for diagnosing obesity: underweight (BMI < 18.5 kg/m^2^), normal (BMI ≥ 18.5 kg/m^2^, < 23 kg/m^2^), overweight (BMI ≥ 23 kg/m^2^, < 25 kg/m^2^), and obese (BMI ≥ 25 kg/m^2^) according to the International Obesity Task Force for adults in Asian and Pacific regions [27]. In the KNHANES, blood was collected from participants to test for blood lipid profile, total cholesterol, HDL-C, and TG. Dyslipidemia was defined as elevated cholesterol level (hypercholesterolemia: blood total cholesterol, ≥240 mg/dL) or TG level (hypertriglyceridemia: blood TG, ≥200 mg/dL), or lower HDL-C level (hypoHDL-cholesterolemia: blood HDL-C, < 40 mg/dL in men and < 50 mg/dL in women) [28].

### Assessment of other variables

Sociodemographic variables such as age, sex, household income, and education; and lifestyle variables such as regular alcohol consumption, current smoking status, and physical activity were obtained through structured questionnaires. Household income was divided into four categories: low (first quartile), middle-low (second quartile), middle-high (third quartile), and high (fourth quartile). Also, educational level was divided into four categories: elementary school and lower, middle school, high school, and college and higher. Regular alcohol consumption was divided into two categories: ‘yes’ (drank more than once a month over the past year) and ‘no’. Current smoking status was divided into two categories: ‘yes’ (smoked > 100 cigarettes over lifetime and still smoking) and ‘no’. Physical activity was also divided into two categories: ‘active’ (performed moderate-intensity physical activity which requires a moderate amount of effort and causes slightly rapid breathing for ≥30 min once for ≥5 days a week) and ‘inactive’ [4, 29].

### Assessment of dietary total antioxidant capacity

The dietary intake was assessed using a one-day 24-h dietary recall (DR) method. Nutrients intake was calculated based on KNHANES nutrients database. We additionally estimated participants’ dietary TAC using the TAC database developed previously [30]. In brief, the TAC database was developed for 42 dietary antioxidants (i.e., vitamins C and E, carotenoids, and flavonoids), expressed as vitamin C equivalents (VCE) for foods commonly consumed in Korea. Daily TAC (mg VCE) from individual food item was determined by multiplying the daily consumption of the item (g) by the TAC of each food (mg VCE/100 g) in the TAC database. Daily TAC from diet was the sum of the daily TAC from all the food items reported in the 24-h DR.

### Dietary pattern analysis

In total, 838 foods were reported in this study, and they were categorized into 24 food groups based on the Korean Nutrition Database and previous studies on the dietary patterns in Korea [31, 32] (Additional file 1: Table S1). Since rice is the staple food of Koreans, it was separated solely from the grain group. Similarly, since Kimchi is a most typically and frequently consumed Korean food, it was also separated from vegetables and seasoning group. Kimchi is representative Korean fermented food known for its unique spicy taste and its health benefit related to antibacterial, anti-inflammatory, and anticancer potential [33–37], and recently introduced and consumed in many countries around the world. Meat was grouped as ‘red meat and its products’ and ‘white meat and its product’, on the basis of the nutrients content and the association of the food group with chronic diseases. Also, vegetable was categorized into ‘green and yellow vegetables’ and ‘light-colored vegetables’ since green and yellow vegetables generally contain more antioxidant compounds than light-colored vegetables.

The average energy intake from the 24 food groups was calculated individually. We conducted an explanatory factor analysis to identify the major dietary pattern based on the percentage of the total daily energy intake from the 24 food groups. In order to determine the number of factors, we considered eigenvalues (> 1.20) and the scree plot (Additional file 2: Figure S1). The derived patterns were named from foods loaded most positively on the pattern. Participants had their own factor score for identified patterns, and they were categorized into tertiles for every emerged pattern by subject’s factor score.

### Statistical analysis

All statistical analyses were performed in SAS version 9.4 (SAS Institute Inc., Cary, NC, USA). We conducted the factor analysis using the FACTOR PROCEDURE in SAS 9.4. The factors were rotated by an orthogonal transformation using the Varimax rotation function in SAS to achieve a simpler structure and greater interpretability. The participants were divided into three groups according to each four dietary pattern scores tertiles. Differences across tertile groups of the dietary pattern scores were determined using chi-square test for categorical variables and generalized linear model for continuous variables. The assumption of generalized linear model that the dependent variable is independently distributed was checked by the plots and satisfied in all models. Multivariable-adjusted logistic regression analysis was conducted to compare the odds ratios (OR) and 95% confidence intervals (CIs) for dyslipidemia among tertiles of each dietary pattern score and the nine combination groups (joint classifications for dietary pattern score tertiles and dietary TAC level tertiles). Logistic regression models were adjusted for sex, age, obesity level, household income, regular alcohol consumption, smoking status, physical activity, and energy intake. In general, because the correlation between educational level and household income level was high, only the household income level was selected as adjustment variable. Sensitivity analyses were conducted not only including education level as adjustment variable in the model but also excluding household income level after adjusted for education level. P for trend values were tested by assigning the median of each dietary pattern score as a continuous variable in the logistic regression model and using the CONTRAST statement in the logistic regression model to determine the linear trend for dyslipidemia according to tertiles of four dietary pattern and joint classifications for dietary pattern score tertiles and dietary TAC level tertiles, respectively. In the complex sampling design data, the missing values of the covariates are weighted in the relevant strata, so non-responder information should be reflected in the analysis without missing the information. We treated all missing values as ‘valid values’ in all the analyses. Statistical significance was defined as *P* < 0.05.

## Results

Four dietary patterns were identified from the factor analysis (Table 1), and were named after food or food groups with high factor loading values. These four dietary patterns accounted for 26% of the total variance: 9.0, 6.1, 5.5, and 5.1%, respectively. The “Rice & Kimchi” pattern featured a high consumption of rice and Kimchi whereas it had a negative loading for noodles and dumpling, bread and snack, fruits, eggs, milk, and dairy products; and beverage. The “Oil, sweets, fish & other vegetables” pattern had high positive factor loading for oil, sweets, seasoning, fish, and shellfish; as well as eggs and other vegetables (non-green and yellow vegetables). The “Red meat & alcohol” pattern was characterized by high consumption of red meat and alcohol, while the “Grain, bean, nuts, vegetables, & fruits” pattern showed high positive factor loading for other grains, fruits, bean, Tofu, and soymilk; nuts and seeds; and vegetables including green and yellow vegetables.

**Table 1:** Factor loading matrix of food groups for the four dietary patterns identified from the 24-h dietary recall

Food or food groups	Pattern 1 Rice & Kimchi	Pattern 2 Oil, sweets, seasoning, fish & other vegetables	Pattern 3 Red meat & alcohol	Pattern 4 Grain, bean, nuts, vegetables & fruits
Rice	0.74	−0.25	−0.39	
Other grains				0.32
Noodles and dumpling	−0.29			− 0.39
Bread and snack	−0.58			
Potatoes and corn				
Sweets		0.53		
Seasoning		0.34	0.33	0.28
Bean, Tofu, and soymilk				0.31
Nuts and seeds				0.36
Green and yellow vegetables				0.40
Light-colored vegetables		0.41		0.33
Kimchi	0.34			
Mushroom				0.25
Fruits	−0.27			0.30
Red meats and its product			0.61	
White meats and its product				
Eggs	−0.25	0.26		−0.28
Fish and Shellfish		0.39		
Seaweeds			−0.33	
Milk and dairy products	−0.51			
Oil		0.74		
Beverage	−0.27			
Alcohol			0.65	
Coffee and Tea				−0.33

* For simplicity, factor loading values less than 0.25 are not listed in the matrix.* Four dietary patterns were derived by principal component analysis after entering the 24 food groups into the FACTOR PROCEDURE. The factors were rotated by orthogonal transformations (Varimax rotation function in SAS), and the number of factors was determined based on eigenvalues (> 1.20)

Table 2 shows the general characteristics of study participants according to the four dietary pattern scores. Participants with higher scores of the “Rice & Kimchi” pattern were more likely to be older whereas participants with higher scores of “Oil, sweets, fish, & other vegetables” pattern and “Red meat & alcohol” pattern were more likely to be younger (all *P* < 0.001). The highest pattern score group for “Grain, bean, nuts, vegetables, & fruits” was associated with the highest proportion of women. The “Grain, bean, nuts, vegetables, & fruits” pattern score was inversely related with regular alcohol consumption and current smoking (all *P* < 0.001).

**Table 2:** General characteristics of the study participants according to the four dietary pattern scores

	Rice & Kimchi			Oil, sweets, fish & other vegetables			Red meat & alcohol			Grain, bean, nuts, vegetables & fruits		
N (%)	T1	T2	T3	T1	T2	T3	T1	T2	T3	T1	T2	T3
Sex												
Male	3182 (32.2)	4234 (42.9)	4490 (45.5)^***^	3503 (35.5)	4055 (41.1)	4348 (44.0)^***^	3267 (33.1)	3536 (35.8)	5103 (51.7)^***^	4795 (48.6)	3986 (40.4)	3125 (31.7)^***^
Age (yr)												
19–29	2091 (21.2)	932 (9.4)	369 (3.7)^***^	880 (8.9)	1091 (11.1)	1421 (14.4)^***^	897 (9.1)	941 (9.5)	1554 (15.7)^***^	1381 (14.0)	1164 (11.8)	847 (8.6)^***^
30–49	4512 (45.7)	4061 (41.1)	2580 (26.1)	2680 (27.1)	3843 (38.9)	4630 (46.9)	3441 (34.9)	3524 (35.7)	4188 (42.4)	4298 (43.5)	3646 (36.9)	3209 (32.5)
50–64	2163 (21.9)	2883 (29.2)	3009 (30.5)	2903 (29.4)	2744 (27.8)	2408 (24.4)	2768 (28.0)	2742 (27.8)	2545 (25.8)	2170 (22.0)	2656 (26.9)	3229 (32.7)
65+	1108 (11.2)	1999 (20.2)	3917 (39.7)	3411 (34.6)	2197 (22.3)	1416 (14.3)	2768 (28.0)	2668 (27.0)	1588 (16.1)	2025 (20.5)	2409 (24.4)	2590 (26.2)
Household income^1^												
Low	1156 (11.9)	1742 (17.9)	3069 (31.8)^***^	2749 (28.5)	1879 (19.4)	1339 (13.8)^***^	2242 (23.1)	2178 (22.5)	1547 (15.9)^***^	1992 (20.6)	2024 (20.9)	1951 (20.1)^***^
Middle-low	2254 (23.2)	2445 (25.1)	2679 (27.8)	2560 (26.5)	2496 (25.7)	2322 (23.9)	2529 (26.1)	2423 (25.0)	2426 (24.9)	2551 (26.3)	2510 (25.9)	2317 (23.8)
Middle-high	2992 (30.8)	2790 (28.7)	2073 (21.5)	2272 (23.5)	2632 (27.1)	2951 (30.3)	2544 (26.3)	2485 (25.7)	2826 (29.0)	2695 (27.8)	2612 (26.9)	2548 (26.2)
High	3319 (34.1)	2759 (28.3)	1825 (18.9)	2080 (21.5)	2699 (27.8)	3124 (32.1)	2373 (24.5)	2595 (26.8)	2935 (30.2)	2446 (25.3)	2556 (26.4)	2901 (29.9)
Education												
≤ Elementary school	1200 (12.3)	2391 (24.6)	4500 (46.5)^***^	3916 (40.4)	2553 (26.2)	1622 (16.7)^***^	3125 (32.2)	3045 (31.3)	1921 (19.8)^***^	2348 (24.2)	2860 (29.5)	2883 (29.6)^***^
Middle school	796 (8.2)	1152 (11.8)	1350 (14.0)	1225 (12.6)	1132 (11.6)	941 (9.7)	1135 (11.7)	1098 (11.3)	1065 (11.0)	1041 (10.7)	1069 (11.0)	1188 (12.2)
High school	3912 (40.2)	3418 (35.1)	2476 (25.6)	2733 (28.2)	3344 (34.4)	3729 (38.3)	3127 (32.2)	3026 (31.1)	3653 (37.6)	3588 (37.0)	3188 (32.8)	3030 (31.1)
≥ College	3829 (39.3)	2778 (28.5)	1350 (14.0)	1822 (18.8)	2701 (27.8)	3434 (35.3)	2323 (23.9)	2554 (26.3)	3080 (31.7)	2719 (28.0)	2592 (26.7)	2646 (27.2)
Regular alcohol consumption^2^												
Yes	4891 (50.4)	5248 (54.0)	4750 (49.0)^***^	4235 (43.7)	5166 (53.2)	5488 (56.5)^***^	3952 (40.7)	4352 (44.8)	6585 (67.9)^***^	5745 (59.4)	4971 (51.2)	4173 (42.9)^***^
Current smoking^3^												
Yes	2116 (21.7)	2800 (28.8)	3061 (31.6)^***^	2333 (24.1)	2656 (27.3)	2988 (30.7)^***^	2236 (23.0)	2261 (23.2)	3480 (35.8)^***^	3681 (38.0)	2518 (25.9)	1778 (18.2)^***^
Physical activity^4^												
Yes	1072 (11.0)	1122 (11.6)	1172 (12.1)	1187 (12.3)	1162 (12.0)	1017 (10.5)^**^	1111 (11.5)	1106 (11.4)	1149 (11.8)	1078 (11.1)	1117 (11.5)	1171 (12.0)
Obesity (Body Mass Index ≥25 kg/m^2^)	2773 (28.2)	3184 (32.4)	3311 (33.7)^***^	3073 (31.3)	3045 (31.0)	3150 (32.0)	3128 (31.8)	3029 (30.8)	3111 (31.6)	3102 (31.6)	3049 (31.0)	3117 (31.7)^**^
Blood lipid profile (Mean ± SD)												
Total cholesterol	186.9 ± 35.8	188.7 ± 36.1	190.1 ± 37.2^***^	189.4 ± 37.3	188.1 ± 36.1	188.2 ± 35.7^*^	189.7 ± 36.7	188.3 ± 36.4	187.7 ± 35.9^***^	189.1 ± 36.4	188.1 ± 36.1	188.5 ± 36.6
HDL cholesterol	53.4 ± 13.1	51.3 ± 12.7	49.5 ± 12.5^***^	50.8 ± 12.8	51.8 ± 13.0	51.6 ± 12.7^***^	51.0 ± 12.8	50.9 ± 12.7	52.4 ± 13.0^***^	51.7 ± 12.9	51.2 ± 12.8	51.4 ± 12.9
Triglyceride	117.4 ± 88.7	133.2 ± 107.4	142.4 ± 103.0^***^	136.8 ± 108.5	129.1 ± 95.8	127.2 ± 96.7^***^	128.8 ± 94.2	127.5 ± 92.5	136.8 ± 113.4^***^	137.4 ± 114.2	129.8 ± 96.5	125.9 ± 88.9^***^

* The cutpoint of dietary pattern scores for T1 and T2 was −0.3 and 0.5 in the “Rice & Kimchi” pattern, −0.5 and 0.3 in the “Oil, sweets, fish & other vegetables” pattern, −0.5 and 0.1 in the “Red meat & alcohol” pattern, and − 0.4 and 0.3 in the “Grain, bean, nuts, vegetables & fruits” pattern, respectively

* *P* values were calculated by chi-square test except for blood lipid profile; P values for blood lipid profile were calculated by generalized linear model (GLM) ^*^ P < 0.05, ^**^P < 0.01, ^***^P < 0.001

^1^ Household income: low (first quartile), middle-low (second quartile), middle-high (third quartile), high (fourth quartile)

^2^ Regular alcohol consumption: ‘yes’ meant drank more than once a month over the past year

^3^ Current smoking: ‘yes’ meant smoked > 100 cigarettes over lifetime and still smoking

^4^ Physical activity: ‘active’ meant performed moderate-intensity physical activity which requires a moderate amount of effort and causes slightly rapid breathing for ≥30 min once for ≥5 days a week

^1–4^ Number of missing values was 521, 472, 519, 461, and 510 for household income, education level, regular alcohol consumption, current smoking status, and physical activity, respectively

Nutrients intake and dietary antioxidant capacity of participants, according to the four dietary pattern scores are shown in Table 3. Because the nutrient intake and dietary antioxidant capacity were not normally distributed, the medians (25th – 75th percentiles) for each nutrient and antioxidant were presented. The “Rice & Kimchi” pattern score was inversely associated with total energy, vitamin C, vitamin E, carotenoids, flavonoids, and dietary TAC (all *P* < 0.001 ). On the other hand, “Oil, sweets, fish, & other vegetables” pattern and “Red meat & alcohol” pattern were positively associated with total energy, vitamin C, vitamin E, carotenoids, flavonoids, and dietary TAC (all *P* < 0.05). The difference of dietary TAC intake between tertiles of pattern score was largest in the “Grain, bean, nuts, vegetables, & fruits” pattern (the lowest tertile: median [25th - 75th percentiles], 138.7 [73.4–267.2] mg/day; the highest tertile: 358.6 [198.0–677.2] mg/day, *P* < 0.001 ).

**Table 3:** Nutrients intake and dietary antioxidant capacity of participants according to the four dietary pattern scores

	Tertile of pattern scores			
Median (25th - 75th percentiles)	T1	T2	T3	*p*-value
Rice & Kimchi				
Energy (Kcal/d)	1880.3 (1444.7–2433.4)	1852.7 (1419.5–2368.7)	1583.8 (1235.4–2007.7)	< 0.001
Dietary total antioxidant capacity				
Vitamin C (mg VCE/d)	77.9 (35.1–173.0)	71.5 (36.8–142.0)	49.0 (24.7–92.3)	< 0.001
Retinol (mg VCE/d)	0.0 (0.0–0.1)	0.0 (0.0–0.1)	0.0 (0.0–0.0)	< 0.001
Vitamin E (mg VCE/d)	4.3 (2.8–6.4)	3.9 (2.6–5.9)	3.0 (1.8–4.6)	< 0.001
Carotenoids (mg VCE/d)	1.5 (0.7–3.5)	1.4 (0.7–3.2)	1.0 (0.4–2.3)	< 0.001
Flavonoids (mg VCE/d)	165.3 (71.1–371.9)	163.0 (79.5–319.4)	104.2 (49.8–212.5)	< 0.001
TAC (mg VCE/d)	273.2 (133.0–576.3)	260.6 (137.1–482.8)	169.7 (89.3–318.5)	< 0.001
Oil, sweets, fish, & other vegetables				
Energy (Kcal/d)	1647.4 (1272.3–2130.5)	1778.6 (1387.1–2265.7)	1869.5 (1419.9–2406.9)	< 0.001
Dietary total antioxidant capacity				
Vitamin C (mg VCE/d)	57.9 (23.3–141.8)	62.6 (32.3–125.1)	70.4 (38.3–128.8)	< 0.001
Retinol (mg VCE/d)	0.0 (0.0–0.0)	0.0 (0.0–0.1)	0.0 (0.0–0.1)	< 0.001
Vitamin E (mg VCE/d)	2.9 (1.8–4.5)	3.6 (2.4–5.3)	4.8 (3.2–6.9)	< 0.001
Carotenoids (mg VCE/d)	0.8 (0.3–2.0)	1.3 (0.6–3.0)	1.8 (0.9–4.0)	< 0.001
Flavonoids (mg VCE/d)	129.9 (51.6–317.4)	142.3 (67.4–296.6)	147.6 (76.8–277.5)	< 0.001
TAC (mg VCE/d)	210.9 (92.5–484.8)	230.0 (120.8–446.6)	241.5 (136.8–426.5)	< 0.001
Red meat & alcohol				
Energy (Kcal/d)	1574.2 (1229.1–1993.2)	1716.7 (1338.0–2180.3)	2056.6 (1570.3–2648.9)	< 0.001
Dietary total antioxidant capacity				
Vitamin C (mg VCE/d)	61.7 (27.0–141.3)	65.3 (31.7–134.3)	64.9 (35.3–121.4)	< 0.001
Retinol (mg VCE/d)	0.0 (0.0–0.1)	0.0 (0.0–0.1)	0.0 (0.0–0.1)	< 0.001
Vitamin E (mg VCE/d)	3.5 (2.2–5.3)	3.6 (2.3–5.5)	4.1 (2.7–6.1)	< 0.001
Carotenoids (mg VCE/d)	1.1 (0.5–2.7)	1.3 (0.5–3.1)	1.4 (0.7–3.2)	< 0.001
Flavonoids (mg VCE/d)	132.6 (54.3–307.0)	143.1 (64.4–306.2)	145.9 (75.6–277.8)	0.019
TAC (mg VCE/d)	220.0 (100.0–470.1)	233.3 (118.2–464.3)	234.1 (129.9–417.4)	< 0.001
Grain, bean, nuts, vegetables, & fruits				
Energy (Kcal/d)	1789.6 (1365.6–2357.2)	1799.7 (1386.4–2305.2)	1706.0 (1320.4–2177.9)	< 0.001
Dietary total antioxidant capacity				
Vitamin C (mg VCE/d)	42.4 (22.2–82.3)	66.0 (33.4–127.1)	96.9 (47.5–194.5)	< 0.001
Retinol (mg VCE/d)	0.0 (0.0–0.1)	0.0 (0.0–0.1)	0.0 (0.0–0.1)	0.632
Vitamin E (mg VCE/d)	3.4 (2.1–5.1)	3.6 (2.3–5.5)	4.3 (2.8–6.4)	< 0.001
Carotenoids (mg VCE/d)	0.9 (0.4–1.8)	1.2 (0.6–2.8)	2.0 (0.9–4.7)	< 0.001
Flavonoids (mg VCE/d)	82.6 (37.6–171.0)	143.2 (70.1–280.5)	225.1 (114.7–455.6)	< 0.001
TAC (mg VCE/d)	138.7 (73.4–267.2)	230.8 (125.3–426.2)	358.6 (198.0–677.2)	< 0.001

* The cutpoint of dietary pattern scores for T1 and T2 was − 0.3 and 0.5 in the “Rice & Kimchi” pattern, − 0.5 and 0.3 in the “Oil, sweets, fish & other vegetables” pattern, − 0.5 and 0.1 in the “Red meat & alcohol” pattern, and − 0.4 and 0.3 in the “Grain, bean, nuts, vegetables & fruits” pattern, respectively

*P values were calculated by generalized linear model (GLM)

*VCE* vitamin C equivalents, *TAC* total antioxidant capacity

Finally, we examined dyslipidemia risk according to the four dietary pattern scores (Table 4) and the joint classifications of the dietary pattern scores and dietary TAC (Table 5). After adjustment for sex, age, obesity level, household income, regular alcohol consumption, smoking status, physical activity, and energy intake, the OR for risks of hypercholesterolemia, hypertriglyceridemia, and hypoHDL-cholesterolemia from the lowest to the highest tertiles of the “Rice & Kimchi” pattern scores were 0.79 [95% CI 0.72–0.87], 1.33 [95% CI 1.20–1.47], and 1.21 [95% CI 1.13–1.30], respectively. It seemed that the “Rice & Kimchi” pattern had the opposite association with hypertriglyceridemia compared to the “Oil, sweets, fish, & other vegetables” pattern and the “Grain, bean, nuts, vegetables, & fruits” pattern. While the highest tertile of the “Rice & Kimchi” pattern scores showed a 33% higher likelihood of having hypertriglyceridemia compared to the lowest tertile, the highest tertile of the “Oil, sweets, fish & other vegetables” pattern scores and the “Grain, bean, nuts, vegetables, & fruits” pattern scores showed 27 and 12% lower likelihood of having hypertriglyceridemia compared to the lowest tertile, respectively. Also, the “Rice & Kimchi” pattern have the opposite association with hypoHDL-cholesterolemia risk compared to the “Red meat & alcohol” pattern. The highest tertile of the “Red meat & alcohol” pattern scores showed an 12% lower likelihood of having hypoHDL-cholesterolemia compared to the lowest tertile. The results of sensitivity analyses including education level as adjustment variable and excluding household income level after adjusted for education level were similar to those in Table 4 (Data not shown).

**Table 4:** Odds ratios and 95% confidence intervals of dyslipidemia risk according to the dietary pattern score

	Tertile of pattern score							
	Rice & Kimchi				Oil, sweets, fish & other vegetables			
	T1	T2	T3	p for trend	T1	T2	T3	p for trend
Hypercholesterolemia	1118 (11.8)^§^ 1.00	1251 (13.2) **0.90 [0.82–0.99]**	1334 (14.2) **0.79 [0.72–0.87]**	**< 0.001**	1410 (14.9) 1.00	1198 (12.6) 0.96 [0.88–1.05]	1095 (11.6) 0.99 [0.90–1.08]	0.820
Hypertriglyceridemia	884 (11.2) 1.00	1153 (14.5) 1.09 [0.99–1.21]	1476 (18.1) **1.33 [1.20–1.47]**	**< 0.001**	1356 (16.6) 1.00	1148 (14.4) **0.84 [0.77–0.92]**	1009 (12.9) **0.73 [0.66–0.80]**	**< 0.001**
HypoHDL-cholesterolemia	2935 (30.5) 1.00	3137 (32.7) **1.07 [1.00–1.14]**	3730 (38.9) **1.21 [1.13–1.30]**	**< 0.001**	3619 (37.6) 1.00	3130 (32.6) **0.93 [0.87–0.99]**	3053 (31.8) 0.97 [0.91–1.04]	0.469
	Red meat & alcohol				Grain, bean, nuts, vegetables & fruits			
	T1	T2	T3		T1	T2	T3	
Hypercholesterolemia	1380 (14.6) 1.00	1247 (13.2) **0.92 [0.84–1.00]**	1076 (11.4) 0.98 [0.89–1.07]	0.744	1132 (12.1) 1.00	1159 (12.2) **0.88 [0.81–0.97]**	1412 (14.8) 0.97 [0.89–1.07]	0.790
Hypertriglyceridemia	1134 (14.0) 1.00	1132 (13.9) 1.00 [0.91–1.10]	1247 (16.0) 1.09 [0.98–1.20]	0.081	1253 (16.1) 1.00	1156 (14.4) 0.93 [0.85–1.02]	1104 (13.4) **0.88 [0.80–0.97]**	**0.009**
HypoHDL-cholesterolemia	3600 (37.5) 1.00	3588 (37.3) **1.08 [1.01–1.15]**	2614 (27.3) **0.88 [0.83–0.95]**	**< 0.001**	2894 (30.4) 1.00	3319 (34.4) **1.12 [1.05–1.20]**	3589 (37.2) **1.14 [1.07–1.22]**	**< 0.001**

^§^ The number of cases (percentage)

*Figures with bold fonts represent statistical significance. * Adjusted for sex, age, obesity level, household income, regular alcohol consumption, smoking status, physical activity, and energy intake

* hypercholesterolemia meant blood total cholesterol is ≥240 mg/dL; hypertriglyceridemia meant blood triglyceride is ≥200 mg/dL; hypoHDL-cholesterolemia meant blood HDL cholesterol is ≤40 mg/dL in men and ≤ 50 mg/dL in women

*HDL* high-density lipoprotein

**Table 5:** Odds ratios and 95% confidence intervals of dyslipidemia risk according to the joint classifications of the dietary pattern score and dietary total antioxidant capacity

		Tertile of pattern score					
		Rice & Kimchi			Oil, sweets, fish & other vegetables		
		T1	T2	T3	T1	T2	T3
		Hypercholesterolemia			Hypercholesterolemia		
Dietary TAC (mg VCE/d)	T1	1.00	1.00 [0.83–1.20]	0.89 [0.75–1.05]	1.00	1.03 [0.89–1.19]	0.95 [0.80–1.11]
	T2	1.08 [0.90–1.30]	1.02 [0.86–1.21]	0.89 [0.75–1.06]	1.04 [0.90–1.21]	0.96 [0.82–1.11]	1.08 [0.93–1.25]
	T3	1.18 [1.00–1.40]	0.97 [0.82–1.15]	**0.82 [0.68–0.99]**	1.08 [0.94–1.25]	1.01 [0.87–1.18]	1.04 [0.89–1.22]
	p for trend	0.511			0.492		
		Hypertriglyceridemia			Hypertriglyceridemia		
	T1	1.00	1.13 [0.93–1.36]	**1.35 [1.13–1.60]**	1.00	**0.82 [0.71–0.96]**	**0.73 [0.62–0.86]**
	T2	0.93 [0.76–1.13]	0.92 [0.77–1.11]	**1.24 [1.03–1.48]**	0.89 [0.76–1.04]	**0.80 [0.69–0.93]**	**0.62 [0.53–0.73]**
	T3	0.93 [0.78–1.12]	1.09 [0.91–1.30]	1.19 [0.98–1.45]	0.89 [0.77–1.03]	**0.73 [0.63–0.85]**	**0.69 [0.58–0.81]**
	p for trend	**0.020**			**0.042**		
		HypoHDL-cholesterolemia			HypoHDL-cholesterolemia		
	T1	1.00	1.05 [0.93–1.19]	**1.20 [1.07–1.34]**	1.00	0.93 [0.83–1.03]	0.95 [0.85–1.06]
	T2	1.01 [0.90–1.14]	1.00 [0.89–1.13]	**1.19 [1.06–1.34]**	0.99 [0.88–1.11]	0.92 [0.82–1.02]	0.94 [0.84–1.04]
	T3	0.99 [0.88–1.11]	1.14 [1.02–1.28]	**1.27 [1.11–1.45]**	0.99 [0.89–1.11]	0.93 [0.83–1.04]	1.02 [0.91–1.14]
	p for trend	0.708				0.714	
		Red meat & alcohol			Grain, bean, nuts, vegetables & fruits		
		T1	T2	T3	T1	T2	T3
		Hypercholesterolemia			Hypercholesterolemia		
Dietary TAC (mg VCE/d)	T1	1.00	0.92 [0.79–1.06]	0.90 [0.76–1.06]	1.00	**0.79 [0.68–0.91]**	0.91 [0.76–1.08]
	T2	0.95 [0.82–1.11]	0.95 [0.82–1.10]	1.02 [0.87–1.19]	0.93 [0.79–1.08]	0.92 [0.80–1.06]	0.96 [0.84–1.10]
	T3	1.05 [0.91–1.22]	0.90 [0.78–1.05]	1.03 [0.87–1.21]	1.03 [0.85–1.24]	0.90 [0.77–1.04]	0.97 [0.86–1.10]
	p for trend	0.439			0.345		
		Hypertriglyceridemia			Hypertriglyceridemia		
	T1	1.00	**1.20 [1.03–1.40]**	**1.24 [1.06–1.46]**	1.00	**0.86 [0.74–1.00]**	0.84 [0.69–1.01]
	T2	0.97 [0.82–1.15]	0.99 [0.84–1.16]	1.04 [0.89–1.23]	**0.83 [0.71–0.97]**	**0.82 [0.71–0.94]**	**0.82 [0.71–0.95]**
	T3	1.11 [0.95–1.30]	0.92 [0.78–1.08]	1.06 [0.89–1.26]	0.84 [0.70–1.01]	0.90 [0.77–1.04]	**0.80 [0.70–0.91]**
	p for trend	**0.007**			0.104		
		HypoHDL-cholesterolemia			HypoHDL-cholesterolemia		
	T1	1.00	**1.12 [1.01–1.25]**	**0.81 [0.72–0.91]**	1.00	**1.13 [1.02–1.25]**	**1.23 [1.08–1.40]**
	T2	0.97 [0.87–1.08]	1.01 [0.91–1.13]	0.90 [0.80–1.00]	0.96 [0.86–1.07]	1.07 [0.97–1.19]	**1.12 [1.01–1.23]**
	T3	0.99 [0.89–1.10]	1.07 [0.96–1.19]	0.91 [0.81–1.02]	0.99 [0.87–1.13]	**1.12 [1.01–1.25]**	**1.10 [1.01–1.21]**
	p for trend	0.773			0.076		

*Figures with bold fonts represent statistical significance. * Adjusted for sex, age, obesity level, household income, regular alcohol consumption, smoking status and physical activity

* hypercholesterolemia meant blood total cholesterol is ≥240 mg/dL; hypertriglyceridemia meant blood triglyceride is ≥200 mg/dL; hypoHDL-cholesterolemia meant blood HDL cholesterol is ≤40 mg/dL in men and ≤ 50 mg/dL in women

*TAC* total antioxidant capacity, *VCE* vitamin C equivalent, *HDL* high-density lipoprotein

The results of the association between the joint classifications of the dietary pattern scores and dietary TAC, and dyslipidemia risk are presented in Table 5. Among the dyslipidemia components, hypertriglyceridemia was significantly associated with the joint classification of the dietary pattern scores and TAC in all dietary patterns except for the “Grain, bean, nuts, vegetables, & fruits”. In the highest tertile of the “Rice & Kimchi” pattern scores and the “Red meat & alcohol” pattern scores, the lowest tertile of TAC showed 35% and 24% higher likelihood of having hypertriglyceridemia compared to the reference group, respectively. On the other hand, the highest tertile of TAC in the same group showed no significant risk of hypertriglyceridemia. Likewise, in the “Oil, sweets, fish, & other vegetables” pattern, within similar levels of dietary pattern scores, the risk of hypertriglyceridemia was significantly decreased according to the TAC level (p for trend = 0.020, 0.042, and 0.007 for the “Rice & Kimchi” pattern, the “Oil, sweets, fish, & other vegetables” pattern, and the “Red meat & alcohol” pattern, respectively).

## Discussion

In the present study, we derived four distinct dietary patterns, which are, the “Rice & Kimchi”, the “Oil, sweets, fish, & other vegetables”, the “Red meat & alcohol”, and the “Grain, bean, nuts, vegetables, & fruits” patterns, among Korean adults. The major derived patterns were similar to those observed in several other studies [31, 32].

To the knowledge of the authors, this is the first study to identify association between dietary patterns and dietary TAC with dyslipidemia. We found that the “Rice & Kimchi” pattern was associated with low prevalence of hypercholesterolemia and high prevalence of hypertriglyceridemia and hypoHDL-cholesterolemia whereas the “Oil, sweets, fish, & other vegetables” and “Grain, bean, nuts, vegetables, & fruits” patterns were associated with low prevalence of hypertriglyceridemia. The “Red meat & alcohol” pattern was associated with low prevalence of hypoHDL-cholesterolemia. Also, we demonstrated that dietary TAC was inversely associated with the risk of dyslipidemia with a similar pattern score. For all dietary patterns except for the “Grain, bean, nuts, vegetables, & fruits”, dietary TAC was inversely associated with hypertriglyceridemia.

With regard to dyslipidemia, studies on the association between dietary pattern and risk of dyslipidemia were lacking. With blood HDL-C, an earlier study showed that people in countries with high-carbohydrate and low-fat diet tend to have low blood HDL-C [38]. Several studies in Asian countries also demonstrated similar results [39, 40]. In contrast to Western diets, typical Asian diets are rice-based, rich in plant foods, high in carbohydrate, and low in fat [40]. This might be the reason why hypoHDL-cholesterolemia is more prevalent in Asian population than any other country [6]. Also fiber intake from grain, vegetables and fruits was associated with lower blood HDL-C [41]. The results of the present study showing that the “Rice & Kimchi” and “Grain, bean, nuts, vegetables, & fruits” pattern was associated with high prevalence of hypoHDL-cholesterolemia were consistent with findings from a previous study [39–42].

With blood total cholesterol and LDL-C, high intakes of saturated fatty acids and cholesterol directly raise LDL-C concentrations, while the consumption of plant stanols/sterols, viscous (soluble) fiber and soy protein help lower LDL-cholesterol concentrations [43]. Findings from several intervention studies showed that the reduction in dietary total fat, saturated fat, and cholesterol significantly decreased blood LDL-C [44, 45]. Some foods, such as whole grains, vegetables, and some fruits, contain viscous fiber that also helps to lower LDL-C. Previous study indicates that viscous (soluble) forms of dietary fiber can reduce LDL-C levels [43]. High intakes of soy protein can also lower LDL-C levels, especially when it is used to replace animal food products [43]. There is some evidence that the LDL-lowering effect is dependent upon isoflavone content [46], but conclusive data are lacking. Also, low glycemic index (GI) diet was found to have the beneficial effect of lowering LDL-C and total cholesterol as compared with the high GI diet [47]. In this study, we found that the “Rice & Kimchi” pattern was associated with low prevalence of hypercholesterolemia. This is consistent with previous findings that total fat is positively related to the blood total concentration of cholesterol while dietary fiber is inversely associated with the blood LDL-C. However, the “Grain, bean, nuts, vegetables, & fruits” pattern was not linearly associated with hypercholesterolemia.

For blood TG, the evidence suggests that eicosapentaenoic (EPA) and docosahexaenoic (DHA) acids, both omega-3 fatty acids found in fish, lower blood TG [48, 49]. Not only green vegetables but also total vegetable intakes significantly decreased blood TG level [50]. Nut consumption also improved blood TG levels among participants with higher blood TG levels [51]. These findings were supported by the results of the present study that the “Oil, sweets, fish, & other vegetables” and “Grain, bean, nuts, vegetables, & fruits” patterns were associated with low prevalence of hypertriglyceridemia.

In the present study, dietary TAC was inversely associated with dyslipidemia, especially hypertriglyceridemia. This might be attributed to the antioxidants in foods, which protects LDL against oxidation and enhances tumor surveillance though the immune system [18, 19]. Previous studies reported that dietary TAC is inversely associated with the risk of cardiovascular disease [52], and cancer [53, 54]. Our results suggest that dietary TAC may be an important predictor of dyslipidemia, like other well-known diseases such as cardiovascular disease and cancer.

In this study, we uncovered the synergistic effects between dietary patterns and dietary TAC. We found that the prevalence of disease could vary according to the dietary TAC level even in people who share similar dietary pattern. Therefore, in planning a meal, it is necessary to consider not only qualitative quality such as dietary pattern and food composition but also quantitative quality such as dietary TAC.

The strength of this study is in its analytical methodology, which enabled us to figure out the relationship between the combined dietary patterns and dietary TAC, with dyslipidemia. Previous studies on dietary pattern and disease risk were limited because they solely focused on the dietary pattern and its association with disease. Second, this is the first study to identify the association between dietary patterns and dyslipidemia.

However, some limitations also need to be considered in the interpretation of results. First, because of the cross-sectional design of the KNHANES, we cannot confirm causation, but can make inferences about causal association. Second, there is a possibility that the one-day 24-h DR method may not reflect the subject’s usual intake. Finally, the factor analysis approach involves several arbitrary but important decisions, including the consolidation of food items into food groups, the number of factors to extract, the method of rotation, and even the labelling of the components [55]. Therefore, the dietary pattern approach can be somewhat subjective, and the results may be difficult to replicate in other population. Nevertheless, dietary pattern approach is useful especially when many dietary factors have been demonstrated for disease (e.g. cardiovascular disease, metabolic syndrome, and dyslipidemia) because it goes beyond nutrients and foods, to examine the effects of overall diet [14].

## Conclusions

This study provides basic data for the lipid-lowering effect of dietary TAC and its interaction with dietary pattern. Further study will be needed to investigate the association between dietary TAC and dietary pattern with other diseases such as metabolic syndrome, cardiovascular disease, or cancer.

## Additional file

Additional file 1: **Table S1.** Food grouping used in the dietary pattern analysis. (DOCX 14 kb)

Additional file 2: **Figure S1.** Scree plot of eigenvalues after principal component analysis. (DOCX 23 kb)

## Data Availability

The dataset of the KNHANES is accessible through the official website of the KNHANES (http://knhanes.cdc.go.kr).

## Abbreviations

BMI: Body mass index
CI: Confidence interval
DR: Dietary recall
HDL-C: High-density lipoprotein cholesterol
KNHANES: Korea National Health and Nutrition Examination Survey
LDL-C: Low density lipoprotein cholesterol
OR: Odds ratios
TAC: Total antioxidant capacity
TG: Triglycerides
VCE: Vitamin C equivalents
VLDL: Very low-density lipoproteins
