# Supplementary material for: Dietary pattern, dietary total antioxidant capacity, and dyslipidemia in Korean adults
Source: Nutr J. 2019 Jul 13;18:37. doi: 10.1186/s12937-019-0459-x (PMC6626369; doi:10.1186/s12937-019-0459-x)
Supplement: Supplementary file 1 — Table S1. Food grouping used in the dietary pattern analysis. (DOCX 14 kb) [file 12937_2019_459_MOESM1_ESM.docx]

Supplementary Table. Food grouping used in the dietary pattern analysis

| Food or food group | Food items |
| --- | --- |
| Rice | White rice, brown rice, black rice, glutinous rice, cooked rice, rice cake |
| Other grains | barley, oats, millet, buckwheat, sorghum, etc. |
| Noodles and dumpling | Noodles (wheat flour, buckwheat flour), ramyon, udon, spaghetti, etc. |
| Bread and snack | Wheat, flour, bread, sandwich, cake, pizza, hamburger, pie, cereals, biscuit, cookie, etc. |
| Potatoes and corn | Potato, sweet potato, corn, starch noodle, yam, taro, etc. |
| Sweets | Sugar, honey, syrup, candy, chocolate, jelly, caramel, jam, etc. |
| Seasoning | Soybean paste, soy sauce, red pepper paste, red pepper powder, and other seasoning |
| Bean, Tofu, and soymilk | Soybean, mung bean, kidney bean, red bean, pea, soybean curd (Tofu), soymilk, etc. |
| Nuts and seeds | Walnut, peanut, almond, pine nut, chestnut, sesame, perilla seed, etc. |
| Green and yellow vegetables | Carrot, zucchini, lettuce, perilla leaf, leek, pumpkin, spinach, chard, water parsley, pepper, asparagus, etc. |
| Light-colored vegetables | Cabbage, onion, green onion, garlic, radish, eggplant, bean sprouts, balloon flower, bracken, lotus root, etc. |
| Kimchi | Kimchi (cabbage, young radish, cucumber, green onion, cubed radish) |
| Mushroom | Mushrooms |
| Fruits | Apple, pear, strawberry, persimmon, grape, peach, banana, etc. |
| Red meats and its product | Beef, pork, ham, sausage, bacon |
| White meats and its product | Chicken, duck |
| Eggs | egg, quail's egg |
| Fish and Shellfish | Mackerel, tuna, hairtail, pollack, cod, pacific saury, anchovy, shrimp, squid, small octopus, clam, crab, mussel, etc. |
| Seaweeds | Sea mustard, laver, sea tangle |
| Milk and dairy products | Milk, yogurt, cheese, ice cream |
| Oil | Soybean oil, sesame oil, perilla oil, butter, margarine |
| Beverage | Sugar-sweetened beverages |
| Alcohol | Soju, beer, makgeolli, wine, whisky |
| Coffee and Tea | Coffee, instant coffee, green tea, black tea |
