# Supplementary figures and images for: Dietary pattern, dietary total antioxidant capacity, and dyslipidemia in Korean adults
Source: Nutr J. 2019 Jul 13;18:37. doi: 10.1186/s12937-019-0459-x (PMC6626369; doi:10.1186/s12937-019-0459-x)

Supplementary figure. Scree plot of eigenvalues after principal component analysis.


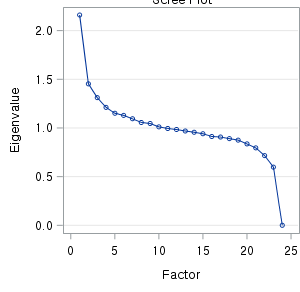

Supplement: Supplementary file 2 — Figure S1. Scree plot of eigenvalues after principal component analysis. (DOCX 23 kb) [file 12937_2019_459_MOESM2_ESM.docx]
